# Samuel John Hutchinson, M.R.C.S., L.D.S. (Eng.)

**Published:** 1905-01

**Authors:** 


### Obituary.

### SAMUEL JOHN HUTCHINSON, M.R.C.S., L.D.S. (ENG.).

MR. HUTCHINSON was born at Helmsley, in Yorkshire, in 1848. He obtained his early education at a local school, and afterwards became a pupil of the late Mr. Jonathan Hooten, of Manchester, where he earned the reputation of being both clever and industrious. This character was maintained upon his arrival in London, where he pursued his studies at University and King's College Hospitals and the Dental Hospital of London. In 1872 he obtained the L.D.S. (Eng.), and two years later the M.R.C.S.

He was one of the early students of the Dental Hospital of London, then in Soho Square, and subsequently became assistant surgeon and surgeon to that institution. He resigned his appointment on becoming lecturer on Dental Surgery and Pathology, which post he again resigned on being elected one of the examiners in Dental Surgery to the Royal College of Surgeons of England. At the date of his death he was consulting dental surgeon to the National Dental Hospital and the University College Hospital.

Mr. Hutchinson may be said to have filled every post of trust and honor that the profession had to offer. In the Odontological Society of Great Britain he was successively member of council, honorary secretary, curator, president, and treasurer. To the

British Dental Association he also gave freely of his time and talents, and had filled the post of chairman of the representative board. He was president of the association at its last annual general meeting in London in 1901. He was also a trustee of the benevolent fund.

His commanding position in the profession was obtained by tireless energy and dogged perseverance. Notwithstanding the cares of a large private practice, he constantly took an active part in his profession's public affairs, and faithfully discharged all the duties of the many offices he held.

His health was at no time robust, and it is possible that he was at times overtaxed. Some years ago he suffered from a severe attack of tachycardia, from which he appeared to have made a complete recovery; recently, however, signs of valvular incompetence became apparent, and rapidly increased, and to this he finally succumbed.

He died at his residence, "Ford," Branksome Park, Bournemouth, September 15, aged fifty-six, and was buried on September 20, 1904, at Hampstead Cemetery.

He left an estate valued at over one hundred and fifty thousand dollars. His will provides that should his children die without leaving issue, the Dental Hospital of London, Leicester Square, and the North London or University College Hospital, will each receive five thousand dollars; the Dental Benevolent Fund of the British Dental Association, the Epsom College Benevolent Fund, and the Society for the Relief of Widows of Medical Men, in the same contingency are to receive five hundred dollars each. (The *Dental Record*, and the *British Journal of Dental Science.*)

### LADY TOMES.

Another link with the past is severed by the decease of Jane, widow of the late Sir John Tomes, F.R.S. Lady Tomes passed away on October 16, 1904, at the ripe age of eighty-one, at her beautiful home, Upwood Gorse, Caterham Valley, England. (*Dental Record.*)

